# Maternal Obesity and Neonatal Death in Preterm US Pacific Islander Neonates Using 2 Analytic Approaches

**DOI:** 10.1001/jamanetworkopen.2025.28924

**Published:** 2025-08-26

**Authors:** Bohao Wu, Sarah Taylor, Nicola L. Hawley, Veronika Shabanova

**Affiliations:** 1 Department of Chronic Disease Epidemiology, Yale School of Public Health, New Haven, Connecticut; 2 Department of Epidemiology, Columbia University Mailman School of Public Health, New York, New York; 3 Department of Pediatrics, Yale School of Medicine, New Haven, Connecticut; 4 Department of Biostatistics, Yale School of Medicine, New Haven, Connecticut

## Abstract

**Importance:**

Owing to a high prevalence of obesity, Pacific Islander individuals in the US are at higher risk for preterm birth (PTB), but outcomes after PTB remain understudied. Existing literature suggests that associations between prepregnancy obesity and neonatal mortality stratified by gestational age (GA) are modest or null, which may be a result of overlooked stratification bias.

**Objective:**

To estimate the association between prepregnancy body mass index (BMI) and neonatal death (NND) following PTB by different degrees of prematurity in US Pacific Islander neonates using birth-based and fetuses-at-risk (FAR) approaches.

**Design, Setting, and Participants:**

This population-based cohort study used 2014 to 2018 data files from the National Center for Health Statistics for Pacific Islander singletons born at 22 to 41 weeks’ GA without congenital anomalies. The 2 at-risk populations were PTBs (for the birth-based approach) and all identified pregnancies resulting in live birth (for the FAR approach). Analyses were finished in March 2023.

**Main Outcomes and Measures:**

The primary outcome was NND following PTB stratified by GA intervals (22-27 weeks, 22-31 weeks, and 22-36 weeks). Cox proportional hazards models were used and stratified by GA at birth.

**Results:**

Among 55 975 mother-neonate dyads (27 320 [48.8%] female neonates), the mean (SD) maternal age was 27.8 (5.8) years, and the mean (SD) gestational age of all neonates was 38.5 (1.9) weeks. The PTB prevalence was 9.3% (5192 neonates), and the neonatal mortality rate was 20.4 deaths per 1000 PTBs by the birth-based approach and 1.9 deaths per 1000 live-born pregnancies by the FAR approach. Among extreme PTBs (22-27 weeks), using the FAR approach, associations between prepregnancy obesity and NND following PTB were evident for obesity class I (adjusted hazard ratio [aHR], 2.31; 95% CI, 1.12-4.79) and class II (aHR, 2.82; 95% CI, 1.24-6.41). These associations were attenuated using the birth-based approach (obesity class I aHR, 1.33; 95% CI, 0.61-2.87; class II aHR, 1.73; 95% CI, 0.71-4.28).

**Conclusions and Relevance:**

In this cohort study of US Pacific Islander individuals, according to the FAR approach, prepregnancy obesity class I and II were associated with an increased rate of NND following PTB, yet these associations may be overlooked when analyses use the birth-based approach. This is likely because prepregnancy BMI affects GA at birth, which, in turn, may have its own confounded association with NND, leading to stratification bias and attenuated associations between prepregnancy BMI and NND using the birth-based approach. The FAR approach offers an alternative that may highlight important risk factors for key perinatal outcomes.

## Key Points

**Question:**

Is maternal obesity associated with degree-of-prematurity-dependent neonatal death (NND) following preterm birth (PTB) among Pacific Islander neonates?

**Findings:**

In this cohort study of 55 975 mother-neonate dyads, using the birth-based approach, among all PTBs, prepregnancy obesity class I and class II were associated with NND following PTB, but this association disappeared among extreme PTBs, likely as a result of stratification bias. Using the fetuses-at-risk approach corrected this association and underscored the importance of maternal prepregnancy obesity for NND following extreme PTB.

**Meaning:**

These findings suggest that the methodological approach matters where there are intermediate factors (in this case, gestational age) associated with both the risk factor and outcome.

## Teaser

This cohort study examines the association between prepregnancy body mass index and neonatal death following preterm birth by different degrees of prematurity in US Pacific Islander neonates using birth-based and fetuses-at-risk approaches.

## Introduction

Between 2014 and 2018, although the US neonatal mortality rate declined from 2.3 deaths per 1000 live births (singletons, no congenital abnormalities) to 2.2 deaths per 1000 live births, the incidence of preterm birth (PTB; <37 weeks’ gestation) increased from 7.6% to 8.1%.^1^ Because PTB is the leading cause of neonatal death (NND; <28 days of life),^2,3^ understanding the epidemiology of NND following PTB, especially among populations affected by both, is important. The prevalence of PTB, neonatal mortality rate, and neonatal mortality rate following PTB are all higher among Pacific Islander women than White women.^1^ Importantly, US Pacific Islander women have higher obesity prevalence compared with White women,^4^ putting them at greater risk of PTB and NND.^5,6^ However, our understanding of the association between maternal obesity and NND, stratified by degree of prematurity, is limited.

Two analytical approaches estimate the associations between perinatal risk factors and birth outcomes: birth-based and fetuses-at-risk (FAR). The major difference is the population considered at risk. The birth-based approach uses only neonates born within a specific gestational age (GA) range to estimate risk, whereas FAR allows for PTB as an intermediate outcome between a risk factor and a postbirth outcome of interest and, thus, uses both PTBs and all ongoing pregnancies as the at risk population.^7^ Using the birth-based approach, paradoxical phenomenon have been reported—for example, among all PTBs, Black infants have higher neonatal mortality than White infants (28.9 vs 19.2 deaths per 1000 PTB), yet when stratified by GA at birth, Black infants born extremely preterm are estimated to have lower neonatal mortality than their White counterparts (230.9 vs 250.6 deaths per 1000 PTBs).^1^ This paradox likely reflects stratification bias,^8^ which creates biased GA-specific estimates owing to unmeasured confounding in the association between GA at birth and outcome. Mathematically, this bias can be overcome with the FAR approach, which correctly estimates that the neonatal mortality rate among Black infants born extremely preterm is almost 3 times the rate among extremely preterm White infants (2.4 vs 0.9 deaths per 1000 ongoing pregnancies).^1^ In other studies, the risk of necrotizing enterocolitis among preterm Black infants was underestimated compared with White infants using the birth-based approach, but not the FAR approach^9^; also, counterintuitively protective, null, or attenuated associations between advanced maternal age and infant mortality have been observed among extreme PTBs using the birth-based approach and the expected high risk of infant mortality observed using FAR.^10^

Most studies examining maternal body mass index (BMI; calculated as weight in kilograms divided by height in meters squared) and postbirth outcomes have used a birth-based approach.^11^ We hypothesize, however, that this association may be subject to stratification bias. Indeed, in a Swedish study,^11^ the association between prepregnancy BMI and infant mortality was attenuated for very vs moderate PTB. Because properly addressing stratification bias requires causal inference methods,^12,13,14^ we examined the association between prepregnancy BMI and NND following PTB among Pacific Islander individuals using both analytical approaches. Additional factors associated with risk of NND following PTB among US Pacific Islander individuals were explored as a secondary aim.

## Methods

### Study Design and Participants

We conducted a population-based retrospective cohort study using 2014 to 2018 live birth cohort data linked to birth-infant death data files (NNDs between 2014 and 2019) from the US National Center for Health Statistics.^15^ We chose 2014 as the starting year since GA was not measured by obstetric estimate^16^ until 2014. We excluded more recent data to limit potential effects of COVID-19. Mother-neonate dyads with maternal race listed as Native Hawaiian or Pacific Islander (61 579 dyads) were included. Neonates with GA less than 22 or more than 41 weeks (309 neonates), plural births (1529 neonates), and those with congenital anomalies (2683 neonates) were excluded. Records missing all variables except maternal race (1083 records [1.8%]) were also excluded (eFigure 1 in Supplement 1). Per Yale University institutional review board policy, no ethical approval or consent was required for use of deidentified public data, in accordance with 45 CFR §46. We followed the Strengthening the Reporting of Observational Studies in Epidemiology (STROBE) reporting guidelines.

### Demographics and Prenatal Characteristics

Demographic and prenatal characteristics were obtained from birth and death certificates. Because there were few NNDs after 27 gestational weeks, neonates were categorized as being born at 22 to 27, 22 to 31, and 22 to 36 weeks on the basis of World Health Organization categories.^2^ GA was determined by obstetric estimate. Prepregnancy BMI was classified as underweight (<18.5), healthy weight (18.5-24.9), overweight (25.0-29.9), obesity class I (30.0-34.9), obesity class II (35.0-39.9), and obesity class III (≥40.0).^17^ Other characteristics are described in eAppendix 1 in Supplement 1.

### Outcomes and Causal Inference Framework

The primary outcome was NND following PTB, defined as death before 28 days of life in a live birth at less than 37 gestational weeks.^2,18^ To illustrate how stratification bias could impact our research question, we used a directed acyclic graph (DAG),^12,19^ in which prepregnancy BMI may directly affect NND following PTB and indirectly through GA at birth. Even after conditioning on all observed covariates that could confound the association between GA at birth and NND, as well as between prepregnancy BMI and NND, there is at least 1 unmeasured variable (Figure). When stratifying on GA at birth and using the birth-based approach, the resulting risk ratio combines the direct effect of prepregnancy BMI on NND and bias resulting from the unmeasured common causes of GA at birth and NND. Empirically, if an association between prepregnancy BMI and NND exists among all preterm infants but is either attenuated, null, or even paradoxically reversed for the gestational strata where birth rate is affected by exposure (eg, more infants are born to mothers with obesity class I than with healthy weight), then the FAR-based risk ratio should be able to adjust for that because it is the product of the birth-based risk ratio and the ratio of birth rates in the exposed (eg, obesity class I) and unexposed (eg, healthy weight).^14,20^ For our primary research question, extending the FAR approach to a postnatal outcome is appropriate since neonatal mortality’s pathogenesis may be established in utero, affecting risk of birth, survival, and growth restriction.^21^

**Figure.**
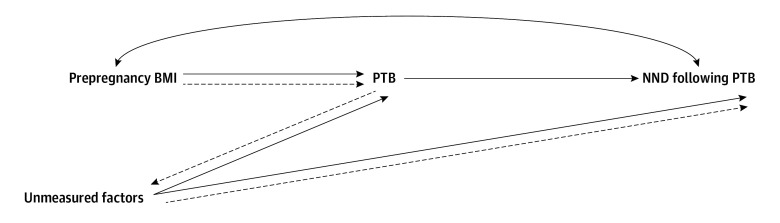
Directed Acyclic Graph Showing Potential Links Between Prepregnancy Body Mass Index (BMI), Preterm Birth (PTB), Neonatal Death (NND) After PTB, and Unmeasured Factors Dashed arrows represent a biased backdoor association of prepregnancy BMI and NND through unmeasured factors when stratified by PTB.

### Birth-Based and FAR Approaches

Birth-based and FAR approaches were characterized by their own at-risk populations and censoring mechanisms. For example, when examining outcomes among those born at 22 to 27 gestational weeks (extreme PTBs), the birth-based approach included only neonates of 22 to 27 gestational weeks at birth in the denominator (at-risk population). In the FAR approach, all neonates born at 22 to 27 weeks of gestation and ongoing pregnancies were included in the denominator. Therefore, in our birth-based models, we censored 22- to 27-week neonates without mortality (birth-based cohort without NND); in FAR, we censored 22- to 27-week neonates without mortality and ongoing pregnancies (FAR cohort without NND). The numerator in both was NNDs in the 22 to 27 GA category.

### Statistical Analysis

We described PTB prevalence, overall neonatal mortality rate, and neonatal mortality rate following PTB. Comparing groups with or without mortality, using both approaches, we examined distribution of prenatal characteristics and birth outcomes. χ^2^ or Fisher exact tests were used to assess between-group differences in categorical characteristics. Continuous characteristics were compared using the Wilcoxon rank-sum test.

Cox proportional hazards regression models, stratified by GA, were used to examine associations between prenatal characteristics and NND following PTB. In these models, the rate (hazard) of NND depends on the observational time and multiplicatively on the effect of a covariate, such that the rate ratio (hazard ratio [HR]) between 2 levels of a covariate (comparison group or reference group) is constant across time. Interactions between covariates and time were tested to evaluate the proportional hazards assumption. For the birth-based approach, the observation time was from live birth to 28 postnatal days; in the FAR approach, it was from conception to postnatal 28 days (gestational weeks plus postnatal days). In each approach, we first selected covariates on the basis of extant literature, then retained covariates from unadjusted models in multivariable models at a 2-sided α = .10. Birth weight was not retained because it is highly correlated with GA and on the same causal pathway. Unadjusted and adjusted hazard rate ratios (aHRs) with 95% CIs were summarized. Presenting findings for all 3 GA groups, we focus on extreme PTBs (22-27 weeks) and all PTBs (22-36 weeks) to highlight differences in our findings by analytic approach. Because the sample size represented a census of pregnancies and births meeting inclusion criteria, statistical inference based on hypothesis tests may not hold to a 2-sided α = .05. Therefore, findings were considered meaningful on the basis of magnitude of effect sizes (HR ≥1.5 or HR ≤0.67) and coverage of 95% CIs, including effect sizes being overwhelmingly in 1 direction. Analyses (completed March 2023) used R version 3.6.2 (R Project for Statistical Computing), RStudio version 2021.09.1 build 372 (RStudio, Inc), and SAS statistical software version 9.4 (SAS Institute).

## Results

Among 55 975 Pacific Islander live-born singletons without congenital anomalies (27 320 [48.8%] female), the mean (SD) maternal age was 27.8 (5.8) years, and the mean (SD) gestational age of all neonates was 38.5 (1.9) weeks. PTB prevalence was 9.3% (5192 neonates), the neonatal mortality rate was 20.4 deaths per 1000 PTBs, and the neonatal mortality rate following PTB was 1.9 deaths per 1000 pregnancies. Among all PTBs (22-36 weeks) we identified 106 NNDs; 5086 neonates were in the birth-based cohort without NND, and 55 869 were in the FAR cohort without NND (Table 1). Results stratified by degree of prematurity are shown in eTables 1 and 2 in Supplement 1. Five-minute APGAR (appearance, pulse, grimace, activity, and respiration) scores and mode of birth differed between NNDs and the other 2 groups, with more NNDs having low (0-3) APGAR scores and being born by cesarean delivery.

**Table 1.** Characteristics of Pacific Islander Mother-Neonate Dyads in the US, 2014-2018, Stratified and Compared by NND

Variable	NND (GA 22-36 wk), No. (%)	No NND (birth based)^a^		No NND (fetuses at risk)^a^	
		No. (%)	*P* value vs group with NND	No. (%)	*P* value vs group with NND
Total	106 (100.0)	5086 (100.0)	NA	55 869 (100.0)	NA
Neonate characteristics					
Sex					
Female	41 (38.7)	2387 (46.9)	.09	27 279 (48.8)	.04
Male	65 (61.3)	2699 (53.1)		28 590 (51.2)	
GA, median (IQR), wk	26.6 (23.4-29.7)	34.2 (32.5-35.9)	<.001	38.5 (37.3-39.8)	<.001
GA categories					
Extreme PTB (22 0/7 to 27 6/7 wk)	71 (67.0)	196 (3.9)	<.001	196 (0.4)	NA
Very PTB (28 0/7 to 31 6/7 wk)	10 (9.4)	414 (8.1)		414 (0.7)	
Moderate-to-late PTB (32 0/7 to 36 6/7 wk)	25 (23.6)	4476 (88.0)		4476 (8.0)	
Full-term birth (37 0/7 to 41 6/7 wk)	NA	NA		50 783 (90.9)	
Birth weight categories by weight					
Median (IQR), g	1145.2 (545.5-1745.0)	2498.2 (2015.7-2980.7)	<.001	3320.6 (2934.7-3706.4)	<.001
Low birth weight (<2500 g)	94 (88.7)	2328 (45.8)	<.001	3543 (6.3)	<.001
Normal weight (2500-4000 g)	11 (10.4)	2676 (52.6)		46 872 (83.9)	
Macrosomia (>4000 g)	1 (0.9)	82 (1.6)		5454 (9.8)	
Birth weight categories by size					
Smaller for GA	8 (7.6)	408 (8.0)	.32	5540 (9.9)	.60
Appropriate for GA	86 (81.1)	3833 (75.4)		43 140 (77.2)	
Larger for GA	12 (11.3)	845 (16.6)		7189 (12.9)	
Maternal characteristics					
Ethnicity					
Hawaiian	8 (7.6)	487 (9.6)	.20	5140 (9.2)	.08
Guamanian	6 (5.7)	584 (11.5)		6892 (12.3)	
Samoan	18 (17.0)	883 (17.4)		10 999 (19.7)	
Other Pacific Islander	74 (69.8)	3132 (61.6)		32 838 (58.8)	
Age, median (IQR), y	28.3 (23.8-32.8)	28.6 (24.3-32.8)	.73	27.8 (23.9-31.8)	.42
Age group, y					
<20	12 (11.3)	347 (6.8)	.19	3522 (6.3)	.05
20-34	75 (70.8)	3721 (73.2)		44 353 (79.4)	
≥35	19 (17.9)	1018 (20.0)		7994 (14.3)	
Nativity					
Born in the US	37 (37.0)	1784 (35.1)	.79	20 870 (37.4)	.86
Born outside the US	63 (63.0)	3212 (63.1)		34 221 (61.3)	
Missing	6 (5.7)	90 (1.8)		778 (1.4)	
Marital status					
Married	48 (45.3)	2231 (43.9)	.67	26 088 (46.7)	.82
Unmarried	50 (47.2)	2535 (49.8)		25 959 (46.5)	
Missing	8 (7.5)	320 (6.3)		3822 (6.8)	
Education					
Less than high school	31 (29.3)	1510 (29.7)	.44	14 315 (25.6)	.19
High school graduate	43 (40.6)	1811 (35.6)		20 001 (35.8)	
Some college credit (no degree)	23 (21.7)	1077 (21.2)		12 749 (22.8)	
Associate degree or above	9 (8.5)	688 (13.5)		8804 (15.8)	
Death of prior children	8 (7.6)	110 (2.2)	<.001	900 (1.6)	<.001
Subsequent live birth	67 (63.2)	3459 (68.0)	.29	39 097 (70.0)	.13
Adequacy of Prenatal Care Utilization Index					
Inadequate	35 (33.0)	1895 (37.3)	.32	19 478 (34.9)	<.001
Intermediate	3 (2.8)	288 (5.7)		6148 (11.0)	
Adequate	17 (16.0)	844 (16.6)		18 390 (32.9)	
Adequate plus	51 (48.1)	2059 (40.5)		11 853 (21.2)	
Enrolled in Supplemental Nutrition Program for Women, Infants and Children	44 (41.5)	2345 (46.1)	.35	28 173 (50.4)	.07
Smoking^b^					
Before pregnancy	7 (6.6)	339 (6.7)	.97	3284 (5.9)	.76
During 1st trimester	6 (5.7)	265 (5.2)	.84	2316 (4.1)	.46
During 2nd trimester	4 (3.8)	218 (4.3)	>.99	1839 (3.3)	.78
During 3rd trimester	3 (2.8)	200 (3.9)	>.99	1739 (3.1)	.75
BMI (weight in kilograms divided by height in meters squared)					
Median (IQR)	30.4 (25.6-35.2)	29.7 (24.9-34.6)	.24	29.5 (24.8-34.2)	.13
Underweight (<18.5)	4 (3.8)	126 (2.5)	.63	1026 (1.8)	.31
Healthy weight (18.5-24.9)	21 (19.8)	1245 (24.5)		14 766 (26.4)	
Overweight (25.0-29.9)	28 (26.4)	1508 (29.7)		16 427 (29.4)	
Obesity class I (30.0-34.9)	29 (27.4)	1155 (22.7)		12 462 (22.3)	
Obesity class II (35.0-39.9)	15 (14.2)	600 (11.8)		6594 (11.8)	
Obesity class III (≥40.0)	9 (8.5)	452 (8.9)		4594 (8.2)	
Rate of gestational weight gain (lb/wk, stratified by BMI)^c^					
<10th Percentile	16 (15.1)	725 (14.3)	.90	5583 (10.0)	.18
10th-90th Percentile	78 (73.6)	3839 (75.5)		44 598 (79.8)	
>90th Percentile	12 (11.3)	522 (10.3)		5688 (10.2)	
Prepregnancy diabetes	1 (0.9)	234 (4.6)	.09	886 (1.6)	>.99
Gestational diabetes	3 (2.8)	601 (11.8)	.004	4549 (8.1)	.05
Prepregnancy hypertension	1 (0.9)	221 (4.4)	.09	845 (1.5)	>.99
Gestational hypertension	11 (10.4)	673 (13.2)	.39	3071 (5.5)	.03
Hypertension eclampsia	0 (0.0)	133 (2.6)	.12	412 (0.7)	>.99
Previous preterm birth	16 (15.1)	598 (11.8)	.29	2238 (4.0)	<.001
Steroid treatment	20 (18.9)	802 (15.8)	.39	989 (1.8)	<.001
Chorioamnionitis	5 (4.7)	69 (13.6)	.02	1047 (1.9)	.05
Payment					
Medicaid	64 (60.4)	2775 (54.6)	.03	30 957 (55.4)	.004
Private Insurance	21 (19.8)	1332 (26.2)		15 153 (27.1)	
Self-pay	16 (15.1)	476 (9.4)		4225 (7.6)	
Other	5 (4.7)	503 (9.9)		5534 (9.9)	
Other birth outcomes					
5-min APGAR score					
0-3	56 (52.8)	68 (1.3)	<.001	249 (0.4)	<.001
4-6	26 (24.5)	290 (5.7)		940 (1.7)	
7-8	15 (14.2)	1410 (27.7)		8135 (14.6)	
9-10	4 (3.8)	3283 (64.5)		46 369 (83.0)	
Missing	5 (4.7)	35 (0.7)		176 (0.3)	
Final route and method of birth					
Spontaneous	52 (49.1)	2847 (56.0)	.21	37 720 (67.5)	<.001
Forceps	1 (0.9)	39 (0.8)		410 (0.7)	
Vacuum	0 (0.0)	80 (1.6)		1277 (2.3)	
Cesarean	53 (50.0)	2119 (41.6)		16 457 (29.5)	
Missing	0 (0.0)	1 (<0.01)		5 (<0.01)	

Abbreviations: APGAR, appearance, pulse, grimace, activity, and respiration; BMI, body mass index; GA, gestational age; NA, not applicable; NND, neonatal death; PTB, preterm birth.

^a^ Corresponding to censored observations that did not experience NND as described in the Methods section.

^b^ Missing data on labeled characteristics are not indicated but are below 0.5%.

^c^ Since the data files do not have information for weight gain in each trimester, the rate of gestational weight gain was classified within prepregnancy BMI categories (described in the Methods section).

### Prepregnancy BMI and NND Following PTB

Using the birth-based approach, among all PTBs, neonates born to mothers with obesity class I (aHR, 1.85; 95% CI, 1.05-3.29) or class II (aHR, 1.97; 95% CI, 0.99-3.89) had higher risk of NND following PTB compared with those born to mothers with a healthy weight. Among extreme PTBs, no association between maternal prepregnancy BMI and NND following PTB was observed (obesity class I aHR, 1.33; 95% CI, 0.61-2.87; obesity class II aHR, 1.73; 95% CI, 0.71-4.28) (Table 2, eTables 3 and 4, and eFigures 2 and 3 in Supplement 1).

**Table 2.** Multivariable Cox Regression Models of the Association Between Prepregnancy BMI and Neonatal Death Following Preterm Birth Among Pacific Islander Neonates in the US, Using Birth-Based and Fetuses-at-Risk Approaches

Approach and prepregnancy BMI^a^	GA 22 0/7 to 27 6/7 wk		GA 22 0/7 to 31 6/7 wk		GA 22 0/7 to 36 6/7 wk	
	aHR (95% CI)^b^	*P* value	aHR (95% CI)^b^	*P* value	aHR (95% CI)^b^	*P* value
Birth-based approach						
Underweight (<18.5)	1.67 (0.40-6.87)	.48	1.74 (0.55-5.49)	.34	1.68 (0.58-4.93)	.34
Healthy weight (18.5-24.9)	1 [Reference]	NA	1 [Reference]	NA	1 [Reference]	NA
Overweight (25.0-29.9)	1.26 (0.57-2.79)	.57	1.04 (0.53-2.02)	.92	1.22 (0.69-2.16)	.50
Obesity class I (30.0-34.9)	1.33 (0.61-2.87)	.47	1.53 (0.79-2.95)	.21	1.85 (1.05-3.29)^c^	.03^c^
Obesity class II (35.0-39.9)	1.73 (0.71-4.28)	.23	2.03 (0.95-4.37)^c^	.07^c^	1.97 (0.99-3.89)^c^	.05^c^
Obesity class III (≥40.0)	1.41 (0.49-4.12)	.53	1.33 (0.51-3.50)	.56	1.73 (0.78-3.83)	.18
Fetuses-at-risk approach						
Underweight (<18.5)	3.24 (0.91-11.54)^c^	.07^c^	3.16 (1.06-9.45)^c^	.04^c^	2.57 (0.88-7.50)^c^	.09^c^
Healthy weight (18.5-24.9)	1 [Reference]	NA	1 [Reference]	NA	1 [Reference]	NA
Overweight (25.0-29.9)	1.50 (0.72-3.12)	.28	1.15 (0.60-2.21)	.67	1.30 (0.74-2.30)	.36
Obesity class I (30.0-34.9)	2.31 (1.12-4.79)^c^	.02^c^	1.63 (0.85-3.13)	.14	1.86 (1.05-3.30)^c^	.03^c^
Obesity class II (35.0-39.9)	2.82 (1.24-6.41)^c^	.01^c^	2.06 (0.98-4.32)^c^	.06^c^	1.97 (1.00-3.88)^c^	.05^c^
Obesity class III (≥40.0)	2.18 (0.80-5.93)	.13	1.43 (0.55-3.71)	.46	1.80 (0.81-4.01)	.15

Abbreviations: aHR, adjusted hazard ratio; BMI, body mass index; GA, gestational age; NA, not applicable.

^a^ BMI is calculated as weight in kilograms divided by height in meters squared.

^b^ Adjusted for neonatal sex and maternal characteristics (age, education attainment, death of prior children, parity >1, adequacy of prenatal care utilization, Women Infants and Children program enrollment, rate of gestational weight gain, gestational diabetes, prepregnancy hypertension, gestational hypertension, previous preterm birth, steroid treatment, chorioamnionitis, and insurance payment).

^c^ Indicates meaningful findings (described in the Methods section).

Using the FAR approach, among all PTBs, maternal prepregnancy underweight (aHR, 2.57; 95% CI, 0.88-7.50), obesity class I (aHR, 1.86; 95% CI, 1.05-3.30), and obesity class II (aHR, 1.97; 95% CI, 1.00-3.88) were associated with increased risk of NND following PTB. Among extreme PTBs, these associations were also observed (underweight aHR, 3.24; 95% CI, 0.91-11.54; obesity class I aHR, 2.31; 95% CI, 1.12-4.79; obesity class II aHR, 2.82; 95% CI, 1.24-6.41) (Table 2).

Among all PTBs, ratios of birth rates were close to 1.0 for all prepregnancy BMI categories relative to healthy weight, with the exception of a 1.47 times higher birth rate among women with underweight vs healthy weight. Among extreme PTBs, the ratios of birth rates ranged from 1.23 for overweight vs healthy weight, to 1.57 to 1.67 for obesity classes I to III, and 1.93 for underweight vs healthy weight (eTables 5, 6, and 7 in Supplement 1).

### Other Variables and NND Following PTB

Using the birth-based approach, 5 additional variables were associated with NND among all PTBs and only 2 additional variables among extreme PTBs. Using the FAR approach, 9 additional variables were identified among all PTBs and 7 among extreme PTBs (eFigures 2 and 3 and eAppendix 2 in Supplement 1).

## Discussion

To our knowledge, this cohort study is the first to estimate the association between prepregnancy BMI and NND following PTB among US Pacific Islander neonates using a causal inference framework, revealing possible stratification bias in birth-based analysis that was resolved with the FAR approach. In all PTBs, both approaches showed increased risk of NND in mothers with prepregnancy obesity class I and class II compared with those with healthy weight. However, among extreme PTBs, these associations were markedly attenuated in the birth-based approach compared with FAR. These findings indicate that, depending on analytical approach, important risk factors for NND, like maternal prepregnancy BMI, can be overlooked when analyses are stratified by GA. In exploratory analyses of other potential risk factors, the 2 approaches identified some overlapping risk factors for NND but diverged on others.

Given the high prevalence of overweight and obesity among US Pacific Islander individuals,^4^ examining prepregnancy BMI as a risk factor for NND following PTB in this population is important. Because obesity is a well-known risk factor for both PTB^22^ and infant mortality,^5,23^ it should be surprising that the birth-based analysis suggests no increased risk of NND for prepregnancy class I or class II obesity among extreme PTBs. Similar paradoxes for other risk factors^10^ likely result from improper adjustment for an intermediate variable common to both risk factor and outcome, stratification bias being 1 such example of overadjustment.^8^ In our study, there was a known association between maternal prepregnancy BMI and both degree of prematurity (intermediate outcome or variable)^22^ and NND (outcome of interest),^11^ and when we stratified by GA, the association between prepregnancy BMI and NND was attenuated in the birth-based approach. As discussed in our methods, collider stratification bias arises when there is a third factor that causes both PTB and NND. Conditioning on PTB opens the true association between prepregnancy BMI and NND. As such, using the birth-based approach, an attenuated association may be observed among neonates born in an earlier GA for risk factors that are also associated with the timing of PTB.^10^ The FAR approach may be needed to study such risk factors, especially if they play a role in the causal pathways leading to the occurrence of PTB itself.

In our study, the FAR approach identified an association between prepregnancy underweight and NND following PTB, whereas the birth-based approach did not. However, this association is plausible because underweight may reflect undernutrition, an important risk factor for PTB,^24^ and a previous study found that the neonatal mortality rate among neonates born to underweight mothers was 1.25 times the rate among those with healthy prepregnancy BMI (3.64 vs 2.91 per 1000 live births).^25^ Although few Pacific Islander mothers in our sample were classified as underweight, this finding is potentially of public health importance. A larger sample is needed to better understand this association.

No association between class III prepregnancy obesity and NND following PTB was found using either approach. We hypothesize that this may be due to hidden selection bias associated with early (and therefore undocumented) fetal losses among this group, since women with high degree of obesity have lower live birth rates.^23^ A study with a careful documentation of early fetal losses would be needed to examine the broader validity of this finding.

Careful consideration should be given in deciding which analytical approach is more appropriate, as our secondary analyses that examined a range of potential risk factors for NND following PTB among Pacific Islander neonates revealed. Ideally, we recommend that each potential risk factor for NND be examined using its own DAG first, but for illustrative purposes, both approaches were purposely used to highlight the commonalities and divergences in the observed associations. Consistent with previous studies, prior child death was associated with NND among both extreme PTBs and all PTBs, in both analytical approaches.^26^ Similar to our findings for prepregnancy BMI, chorioamnionitis was only identified among all PTBs but not among extreme PTBs using the birth-based approach, but the FAR approach remedied that.^27^ However, the FAR approach may not be appropriate for an exposure that has a spurious association with GA. An example is steroid treatment, for which we do not recommend the FAR analysis, because steroid treatment is not meant to affect GA at birth but is rather used between 24 0/7 and 33 6/7 weeks of gestation for women at risk for preterm delivery within 7 days to help neonates survive after a PTB.^28^ We found several discordant associations between the birth-based and the FAR approaches, as well as between all PTBs and extreme PTBs, such as gestational diabetes, insurance payment, prenatal care, and previous PTB. We present these findings to illustrate the need for further exploration.

We recommend the FAR approach as a solution to avoid overadjustment or stratification bias when encountering the phenomenon of attenuated or counterintuitive associations using the birth-based approach (as has been reported for other phenomena^1,9,10,29^), but not as a consistently superior approach to evaluate risk factors for infant outcomes after a live birth.^30^ Although we advocate for the FAR approach for our primary research aim based on our causal inference framework, this approach may identify associations for exposures that have no causal mechanism for a postnatal outcome.^20^ Therefore, selecting a proper analytical approach depends on the specific research question and the potential collider stratification bias in the causal framework. If a research question is about estimating cause-specific risks and risk ratios within the context of PTB (when selecting a cohort based on GA at birth or stratifying), it is important to examine both (1) whether there is a possibility of the collider stratification bias (using a DAG) and (2) whether there is a difference in the birth rates (and how fast they change) across the GA by the level of exposure (cause of interest).

### Strengths and Limitations

A strength of our study is the US-national level data, with comprehensive coverage of Pacific Islander individuals in the US, alongside rich information on demographic and medical characteristics. GA was measured by obstetric estimate, which is considered more reliable than last menstrual period alone.^31^ There are limitations, though: first, approximately 60% of included mothers were classified as Other Pacific Islander, so ethnicity-specific outcomes may be overlooked. Second, indication for steroid treatment was not recorded (we assumed this was for lung maturation in PTBs). Third, pregnancy loss and stillbirths were not included in the infant death data, which may influence estimates using the FAR approach. Fourth, using an administrative dataset, the reliability and measurement validity of variables depends on the rigor of the original data collection, although the dataset includes clear definitions of variables and transparent methods. Fifth, some important variables (ie, fetal distress, major bleeding, and extrauterine infection) were not available for analysis; these should be considered in prospective studies.

## Conclusions

Among US Pacific Islander, extremely preterm, live-born singletons, the association between prepregnancy obesity class I or II and NND following PTB was attenuated compared with all PTBs using a conventional birth-based approach. Using the FAR approach overcomes this attenuation. Examination of risk factors for NND, such as prepregnancy BMI, should be done carefully when stratifying on the degree of prematurity and causal inference should be considered. In general, if there is a known association between a risk factor, an intermediate variable (eg, degree of PTB) and a neonatal outcome (eg, NND), then the FAR approach should be considered in analysis when stratified by GA.

## Notes

Supplement 1. **eAppendix 1.** Methods for Prenatal Characteristics
eReferences
**eAppendix 2.** Results for Other Variables and NND Following PTB **eTable 1.** Characteristics and Birth Outcomes of Pacific Islander Mother-Neonate Dyads in the US, 2014-2018 (NND With GA 22-27 Weeks), Stratified by NND **eTable 2.** Characteristics and Birth Outcomes of Pacific Islander Mother-Neonate Dyads in the US, 2014-2018 (NND With GA 22-31 Weeks), Stratified by NND **eTable 3.** Unadjusted Hazard Ratios for Risk of Neonatal Death Following Preterm Birth (22-27 Weeks, 22-31 Weeks, 22-36 Weeks) Using Birth-Based Approach **eTable 4.** Unadjusted Hazard Ratios for Risk of Neonatal Death Following Preterm Birth (22-27 Weeks, 22-31 Weeks, 22-36 Weeks) Using Fetuses-at-Risk Approach **eTable 5.** Birth Rates, Birth-Based Effect Estimates, and Fetuses-at-Risk Effect Estimates in US Pacific Islander Individuals (GA 22-27 Weeks) **eTable 6.** Birth Rates, Birth-Based Effect Estimates, and Fetuses-at-Risk Effect Estimates in US Pacific Islander Individuals (GA 22-31 Weeks) **eTable 7.** Birth Rates, Birth-Based Effect Estimates, and Fetuses-at-Risk Effect Estimates in US Pacific Islander Individuals (GA 22-36 Weeks, all PTB) **eFigure 1.** Flowchart of the Sample Selection, 2014-2018 Birth Cohort Birth Linked Infant Death Data Files **eFigure 2.** Adjusted Hazard Ratios for Risk of Neonatal Death Following Preterm Birth Using the Birth-Based Approach **eFigure 3.** Adjusted Hazard Ratios for Risk of Neonatal Death Following Preterm Birth Using the Fetuses-at-Risk Approach

Supplement 2.
Data Sharing Statement
