# Error in Figure 1

**DOI:** 10.1001/jamanetworkopen.2025.38959

**Published:** 2025-09-25

In the Original Investigation titled “Maternal Obesity and Neonatal Death in Preterm US Pacific Islander Neonates Using 2 Analytic Approaches,”^1^ published August 26, 2025, there was an error in Figure 1. The top arrow was double-headed but should have had only 1 arrowhead. This article has been corrected.^1^
