# Supplementary material for: Maternal Obesity and Neonatal Death in Preterm US Pacific Islander Neonates Using 2 Analytic Approaches
Source: JAMA Netw Open. 2025 Aug 26;8(8):e2528924. doi: 10.1001/jamanetworkopen.2025.28924 (PMC12381668; doi:10.1001/jamanetworkopen.2025.28924)
Supplement: Supplement 1. — eAppendix 1. Methods for Prenatal Characteristics eReferences eAppendix 2. Results for Other Variables and NND Following PTB eTable 1. Characteristics and Birth Outcomes of Pacific Islander Mother-Neonate Dyads in the US, 2014-2018 (NND With GA 22-27 Weeks), Stratified by NND eTable 2. Characteristics and Birth Outcomes of Pacific Islander Mother-Neonate Dyads in the US, 2014-2018 (NND With GA 22-31 Weeks), Stratified by NND eTable 3. Unadjusted Hazard Ratios for Risk of Neonatal Death Following Preterm Birth (22-27 Weeks, 22-31 Weeks, 22-36 Weeks) Using Birth-Based Approach eTable 4. Unadjusted Hazard Ratios for Risk of Neonatal Death Following Preterm Birth (22-27 Weeks, 22-31 Weeks, 22-36 Weeks) Using Fetuses-at-Risk Approach eTable 5. Birth Rates, Birth-Based Effect Estimates, and Fetuses-at-Risk Effect Estimates in US Pacific Islander Individuals (GA 22-27 Weeks) eTable 6. Birth Rates, Birth-Based Effect Estimates, and Fetuses-at-Risk Effect Estimates in US Pacific Islander Individuals (GA 22-31 Weeks) eTable 7. Birth Rates, Birth-Based Effect Estimates, and Fetuses-at-Risk Effect Estimates in US Pacific Islander Individuals (GA 22-36 Weeks, all PTB) eFigure 1. Flowchart of the Sample Selection, 2014-2018 Birth Cohort Birth Linked Infant Death Data Files eFigure 2. Adjusted Hazard Ratios for Risk of Neonatal Death Following Preterm Birth Using the Birth-Based Approach eFigure 3. Adjusted Hazard Ratios for Risk of Neonatal Death Following Preterm Birth Using the Fetuses-at-Risk Approach [file jamanetwopen-e2528924-s001.pdf]

Supplemental Online Content

Wu B, Taylor S, Hawley N, Shabanova V. Maternal obesity and neonatal death in preterm US Pacific Islander neonates using 2 analytic approaches. *JAMA Netw Open.* 2025;8(8):e2528924. doi:10.1001/jamanetworkopen.2025.28924

eAppendix 1. Methods for Prenatal Characteristics

eReferences

eAppendix 2. Results for Other Variables and NND Following PTB

eTable 1. Characteristics and Birth Outcomes of Pacific Islander Mother-Neonate Dyads in the US, 2014-2018 (NND With GA 22-27 Weeks), Stratified by NND

eTable 2. Characteristics and Birth Outcomes of Pacific Islander Mother-Neonate Dyads in the US, 2014-2018 (NND With GA 22-31 Weeks), Stratified by NND

eTable 3. Unadjusted Hazard Ratios for Risk of Neonatal Death Following Preterm Birth (22-27 Weeks, 22-31 Weeks, 22-36 Weeks) Using Birth-Based Approach

eTable 4. Unadjusted Hazard Ratios for Risk of Neonatal Death Following Preterm Birth (22-27 Weeks, 22-31 Weeks, 22-36 Weeks) Using Fetuses-at-Risk Approach

eTable 5. Birth Rates, Birth-Based Effect Estimates, and Fetuses-at-Risk Effect Estimates in US Pacific Islander Individuals (GA 22-27 Weeks)

eTable 6. Birth Rates, Birth-Based Effect Estimates, and Fetuses-at-Risk Effect Estimates in US Pacific Islander Individuals (GA 22-31 Weeks)

eTable 7. Birth Rates, Birth-Based Effect Estimates, and Fetuses-at-Risk Effect Estimates in US Pacific Islander Individuals (GA 22-36 Weeks, all PTB)

eFigure 1. Flowchart of the Sample Selection, 2014-2018 Birth Cohort Birth Linked Infant Death Data Files

eFigure 2. Adjusted Hazard Ratios for Risk of Neonatal Death Following Preterm Birth Using the Birth-Based Approach

eFigure 3. Adjusted Hazard Ratios for Risk of Neonatal Death Following Preterm Birth Using the Fetuses-at-Risk Approach

This supplemental material has been provided by the authors to give readers additional information about their work.

© 2025 Wu B et al. *JAMA Network Open*.

eAppendix 1. Methods for Prenatal Characteristics

Birth weight was classified as low (<2500 g), normal (2500-4000 g), or macrosomia (>4000 g);[1] and small-, appropriate-, and large-for-GA (based on the 2017 US birth weight percentiles).[2] Maternal information included ethnicity (Hawaiian, Guamanian, Samoan, Other Pacific Islander), age (<20 years, 20-34 years, >=35 years), nativity (born in/outside the US), marital status (married, unmarried), education (less than high school, high school graduate, some college credit, associate degree or above), death of prior children, parity >1, Adequacy of Prenatal Care Utilization Index[3] (inadequate, intermediate, adequate, adequate-plus), Special Supplemental Nutrition Program for Women, Infants, and Children (WIC) enrollment, smoking history (pre-pregnancy, or during 1st/2nd/3rd trimester), rate of gestational weight gain (GWG, lb/week: total GWG over GA, categorized as <10th percentile, 10th-90th percentile, >90th percentile within pre-pregnancy BMI categories), medical risk factors (pre-pregnancy diabetes, gestational diabetes mellitus (GDM), pre-pregnancy hypertension, gestational hypertension, hypertension eclampsia, previous PTB, steroid treatment, chorioamnionitis), and payment type (Medicaid, private insurance, self-pay, other). Missing values for BMI (n=2,971) and gestational weight gain (n=3,361) were imputed with maximum likelihood estimation;[4] missing values for parity (n=5), WIC enrollment (n=2,015), and Adequacy of Prenatal Care Utilization index (n=2,624) were imputed with linear imputation with rounding method.[5]

eReferences

1. Martin JA, Hamilton BE, Ventura SJ, et al. Births: final data for 2009. Natl Vital Stat Rep 2011;60(1):1-70.
2. Aris IM, Kleinman KP, Belfort MB, et al. A 2017 US reference for singleton birth weight percentiles using obstetric estimates of gestation. Pediatrics 2019;144(1)
3. Kotelchuck M. An evaluation of the Kessner adequacy of prenatal care index and a proposed adequacy of prenatal care utilization index. Am J Public Health 1994;84(9):1414-1420. doi:10.2105/AJPH.84.9.1414
4. Yim C. Imputing Missing Data using SAS®. Accessed July 14, 2022. https://support.sas.com/resources/papers/proceedings15/3295-2015.pdf
5. Allison P. Imputation of Categorical Variables with PROC MI Accessed July 14, 2022. https://support.sas.com/resources/papers/proceedings/proceedings/sugi30/113-30.pdf

eAppendix 2. Results for Other Variables and NND Following PTB

Among all PTBs, both analytical approaches showed increased risk of NND following PTB among male neonates, neonates born to mothers with prior child death and mothers who had chorioamnionitis. Only the birth-based approach showed that neonates born to mothers with GDM, or mothers whose insurance payment methods were private or other (vs Medicaid) had a lower risk of NND following PTB. In the FAR approach, the risk of NND among all PTBs increased in the group with adequate plus (vs. adequate) prenatal care, whose mothers received steroid treatment, prior PTB, and self-pay insurance; while the risk decreased among neonates born to mothers who had parity>1, were enrolled in the WIC program, and had private or other insurance.

Among extreme PTBs, both analytical approaches showed increased risk of NND following PTB among neonates born to mothers who had experienced a prior child death. The birth-based approach additionally identified that male neonates had an increased risk of NND following extreme PTB. In the FAR approach, the risk of NND was increased in extreme PTBs whose mothers had adequate plus prenatal care (vs adequate prenatal care), had taken steroid treatment, had chorioamnionitis, and had previous PTB. Extreme PTBs born to mothers who had parity>1, had GDM, and were enrolled in the WIC program had a lower risk of NND following PTB.

© 2025 Wu B et al. *JAMA Network Open*.

eTable 1. Characteristics and Birth Outcomes of Pacific Islander Mother-Neonate Dyads in the US, 2014-2018 (NND With GA 22-27 Weeks), Stratified by NND

Variable	NND (GA 22-27 weeks)	No NND[a] (Birth based)		No NND[a] (FAR)	
	No. (%) or Median (IQR)	No. (%) or Median (IQR)	P-value[b]	No. (%) or Median (IQR)	P-value[b]
Total	71 (100)	196 (100)		55 904 (100)	
Individual					
Sex					
Female	30 (42.3)	104 (53.1)	.12	27 290 (48.8)	.27
Male	41 (57.8)	92 (46.9)		28 614 (51.2)	
Birth weight categories (II)					
Smaller for GA	5 (7.0)	15 (7.7)	.33	5 543 (9.9)	.34
Appropriate for GA	60 (84.5)	151 (77.0)		43 166 (77.2)	
Larger for GA	6 (8.5)	30 (15.3)		7 195 (12.9)	
Maternal					
Ethnicity					
Hawaiian	6 (8.5)	20 (10.2)	.03	5 142 (9.2)	.09
Guamanian	3 (4.2)	25 (12.8)		6 895 (12.3)	
Samoan	11 (15.5)	47 (24.0)		11 006 (19.7)	
Other Pacific Islanders	51 (71.8)	104 (53.1)		32 861 (58.8)	
Age, median (IQR)	28.1 (23.6-32.7)	28.1 (23.6-32.7)	.75	27.8 (23.9-31.8)	.73
< 20 years	8 (11.3)	18 (9.2)	.63	3 526 (6.3)	.12
20-34 years	50 (70.4)	132 (67.4)		44 378 (79.4)	
>= 35 years	13 (18.3)	46 (23.5)		8 000 (14.3)	
Nativity					
Born in the US	27 (38.0)	81 (41.3)	.74	20 880 (37.3)	.68
Born outside the US	40 (56.3)	109 (55.6)		34 244 (61.3)	
Missing	4 (5.6)	6 (3.1)		780 (1.4)	
Marital status					
Married	27 (38.0)	86 (43.9)	.51	26 109 (46.7)	.20
Unmarried	37 (52.1)	97 (49.5)		25 972 (46.5)	
Missing	7 (9.9)	13 (6.6)		3 823 (6.8)	
Education					
Less than high school	21 (29.6)	48 (24.5)	.27	14 325 (25.6)	.35
High school graduate	30 (42.3)	67 (34.2)		20 014 (35.8)	
Some college credit (no degree)	12 (16.9)	52 (26.5)		12 760 (22.8)	
Associate degree or above	8 (11.3)	29 (14.8)		8 805 (15.8)	
Death of prior children	6 (8.5)	5 (2.6)	.07	902 (1.6)	.001
Subsequent live birth	40 (56.3)	99 (50.5)	.40	39 124 (70.0)	.01
Adequacy of Prenatal Care Utilization Index					
Inadequate	25 (35.2)	53 (27.0)	.59	19 488 (34.9)	<.001
Intermediate	3 (4.2)	7 (3.6)		6 148 (11.0)	
Adequate	9 (12.7)	26 (13.3)		18 398 (32.9)	
Adequate Plus	34 (47.9)	110 (56.1)		11 870 (21.2)	
Enrolled in WIC program	26 (36.6)	74 (37.8)	.87	28 191 (50.4)	.02
Smoked before pregnancy[d]	5 (7.0)	17 (8.7)	.64	3 286 (5.9)	0.61

Smoked during 1st trimester[d]	5 (7.0)	15 (7.7)	.84	2 317 (4.1)	.23
Smoked during 2nd trimester[d]	3 (4.2)	11 (5.6)	.77	1 840 (3.3)	.51
Smoked during 3rd trimester[d]	2 (2.8)	7 (3.6)	1.00	1 740 (3.1)	.68
Body mass index, median (IQR)	30.7 (25.9-35.4)	30.5 (25.7-35.3)	.83	29.5 (24.8-34.2)	.11
Underweight (<18.5)	3 (4.2)	4 (2.0)	.86	1 027 (1.8)	.17
Healthy weight (18.5-24.9)	12 (16.9)	40 (20.4)		14 775 (26.4)	
Overweight (25.0-29.9)	18 (25.4)	53 (27.0)		16 437 (29.4)	
Obesity I (30.0-34.9)	20 (28.2)	49 (25.0)		12 471 (22.3)	
Obesity II (35.0-39.9)	12 (16.9)	29 (14.8)		6 597 (11.8)	
Extreme Obesity III (>=40.0)	6 (8.5)	21 (10.7)		4 597 (8.2)	
Rate of GWG (lb/week, stratified by BMI)[c]					
< 10th percentile	12 (16.9)	40 (20.4)	.74	5 587 (10.0)	.15
10th-90th percentile	52 (73.2)	134 (68.4)		44 624 (79.8)	
> 90th percentile	7 (9.9)	22 (11.2)		5 693 (10.2)	
Pre-pregnancy diabetes	0 (0.0)	7 (5.6)	.20	887 (1.6)	.63
Gestational diabetes	3 (4.2)	14 (7.1)	0.57	4 549 (8.1)	0.23
Pre-pregnancy hypertension	1 (1.4)	16 (8.2)	.05	845 (1.5)	1.00
Gestational hypertension	6 (8.5)	18 (9.2)	.04	3 076 (5.5)	.29
Hypertension eclampsia	0 (0.0)	5 (2.6)	.33	412 (0.7)	1.00
Previous preterm birth	13 (18.3)	18 (9.2)	.04	2 241 (4.0)	<.001
Steroid treatment	13 (18.3)	63 (32.1)	.03	996 (1.8)	.01
Chorioamnionitis	5 (7.0)	10 (5.1)		1 047 (1.9)	.01
Payment					
Medicaid	44 (62.0)	105 (53.6)	.36	30 977 (55.4)	.25
Private Insurance	15 (21.1)	58 (29.6)		15 159 (27.1)	
Self-Pay	8 (11.3)	16 (8.2)		4 233 (7.6)	
Other	4 (5.6)	17 (8.7)		5 535 (9.9)	
Other birth outcomes					
Five-minute APGAR score					
A score of 0-3	37 (52.1)	22 (11.2)	<.001	268 (0.5)	<.001
A score of 4-6	19 (26.8)	54 (27.6)		947 (1.7)	
A score of 7-8	9 (12.7)	97 (49.5)		8 141 (14.6)	
A score of 9-10	1 (1.4)	19 (9.7)		46 372 (82.9)	
Missing	5 (7.0)	4 (2.0)		176 (0.3)	
Final route and method of birth					
Spontaneous	41 (57.8)	59 (30.1)	<.001	37 731 (56.4)	-
Forceps	1 (1.4)	1 (0.5)		410 (0.6)	
Vacuum	0 (0.0)	0 (0.0)		1 277 (1.9)	
Cesarean	29 (40.9)	136 (69.4)		16 481 (24.6)	
Missing	0 (0.0)	0 (0.0)		5 (0.0)	

NND, neonatal death. FAR, fetuses-at-risk. GA, gestational age. GWG, gestational age.
[a] Corresponding to censored cohorts which did not experience NND as described in the Methods.
[b] P-value was calculated compared to neonatal death group.
[c] GA 22-27 weeks indicates 22 0/7 weeks – 27 6/7 weeks.
[d] Missing data on labelled characteristics are not indicated but are below 0.5%.

© 2025 Wu B et al. *JAMA Network Open*.

[e] Since the data files does not have information for weight gain in each trimester, the rate of GWG was classified within pre-pregnancy BMI categories (described in the METHODS).

© 2025 Wu B et al. *JAMA Network Open*.

eTable 2. Characteristics and Birth Outcomes of Pacific Islander Mother-Neonate Dyads in the US, 2014-2018 (NND With GA 22-31 Weeks), Stratified by NND

Variable	NND (GA 22-31 weeks) No. (%) or Median (IQR)	No NND[a] (Birth based) No. (%) or Median (IQR)	P-value[b]	No NND[a] (FAR) No. (%) or Median (IQR)	P-value[b]
Total	81 (100)	610 (100)		55 894 (100)	
Individual					
Sex					
Female	33 (40.7)	285 (46.7)	.31	27 287 (48.8)	.15
Male	48 (59.3)	325 (53.3)		28 607 (51.2)	
Birth weight categories (II)					
Smaller for GA	6 (7.4)	46 (7.5)	.08	5 542 (9.9)	.22
Appropriate for GA	69 (85.2)	460 (75.4)		43 157 (77.2)	
Larger for GA	6 (7.4)	104 (17.1)		7 195 (12.9)	
Maternal					
Ethnicity					
Hawaiian	7 (8.6)	50 (8.2)	.35	5 141 (9.2)	.15
Guamanian	4 (4.9)	63 (10.3)		6 894 (12.3)	
Samoan	14 (17.3)	125 (20.5)		11 003 (19.7)	
Other Pacific Islanders	56 (69.1)	372 (61.0)		32 856 (58.8)	
Age, median (IQR)	28.3 (23.8-32.8)	28.8 (24.5-33.1)	0.49	27.8 (23.9-31.8)	0.54
< 20 years	9 (11.1)	44 (7.2)	.40	3 525 (6.3)	.09
20-34 years	57 (70.4)	432 (70.8)		44 371 (79.4)	
>= 35 years	15 (18.5)	134 (22.0)		7 998 (14.3)	
Nativity					
Born in the US	31 (40.8)	223 (36.6)	.55	20 876 (37.3)	.60
Born outside the US	45 (59.2)	376 (61.6)		34 239 (61.3)	
Missing	5 (6.2)	11 (1.8)		779 (1.4)	
Marital status					
Married	33 (44.6)	263 (43.1)	.79	26 103 (46.7)	.34
Unmarried	41 (55.4)	306 (50.2)		25 968 (46.5)	
Missing	7 (8.6)	41 (6.7)		3 823 (6.8)	
Education					
Less than high school	23 (28.4)	162 (26.6)	.93	14 323 (25.6)	.62
High school graduate	32 (39.5)	233 (38.2)		20 012 (35.8)	
Some college credit (no degree)	17 (21.0)	133 (21.8)		12 755 (22.8)	
Associate degree or above	9 (11.1)	82 (13.4)		8 804 (15.8)	
Death of prior children	6 (7.4)	12 (2.0)	.01	902 (1.6)	.002
Subsequent live birth	48 (59.3)	360 (59.0)	.97	39 116 (70.0)	.04
Summary of Adequacy of Prenatal Care Utilization Index					
Inadequate	26 (32.1)	184 (30.2)	.98	19 487 (34.9)	<.001
Intermediate	3 (3.7)	22 (3.6)		6 148 (11.0)	
Adequate	10 (12.4)	72 (11.8)		18 397 (32.9)	
Adequate Plus	42 (51.9)	332 (54.4)		11 862 (21.2)	
Enrolled in WIC program	32 (39.5)	260 (42.6)	.59	28 185 (50.4)	.05
Smoked before pregnancy[d]	5 (6.4)	44 (7.4)	.75	3 286 (6.1)	.81

© 2025 Wu B et al. *JAMA Network Open*.

Smoked during 1st trimester[d]	5 (6.4)	38 (6.4)	1.00	2 317 (4.3)	.39
Smoked during 2nd trimester[d]	3 (3.9)	31 (5.2)	.79	1 840 (3.4)	.75
Smoked during 3rd trimester[d]	2 (3.4)	24 (4.3)	1.00	1 740 (3.3)	.72
Body mass index, median (IQR)	30.1 (25.3-34.9)	30.0 (25.1-34.8)	.78	29.5 (24.8-34.2)	.33
Underweight (<18.5)	4 (4.9)	16 (2.6)	.65	1 026 (1.8)	.18
Healthy weight (18.5-24.9)	17 (21.0)	139 (22.8)		14 770 (26.4)	
Overweight (25.0-29.9)	20 (24.7)	184 (30.2)		16 435 (29.4)	
Obesity I (30.0-34.9)	21 (25.9)	135 (22.1)		12 470 (22.3)	
Obesity II (35.0-39.9)	13 (16.1)	80 (13.1)		6 596 (11.8)	
Extreme Obesity III (>=40.0)	6 (7.4)	56 (9.2)		4 597 (8.2)	
Rate of GWG (lb/week, by stratified BMI)[c]					
< 10th percentile	12 (14.8)	107 (17.5)	.80	5 587 (10.0)	.32
10th-90th percentile	60 (74.1)	443 (72.6)		44 616 (79.8)	
> 90th percentile	9 (11.1)	60 (9.8)		5 691 (10.2)	
Pre-pregnancy diabetes	0 (0.0)	26 (4.3)	.06	887 (1.6)	.64
Gestational diabetes	3 (3.7)	59 (9.7)	.08	4 549 (8.1)	.14
Pre-pregnancy hypertension	1 (1.2)	45 (7.4)	.03	845 (1.5)	1.00
Gestational hypertension	9 (11.1)	82 (13.4)	.56	3 073 (5.5)	.04
Hypertension eclampsia	0 (0.0)	23 (3.8)	.10	412 (0.7)	1.00
Previous preterm birth	15 (18.5)	74 (12.1)	.11	2 239 (4.0)	<.001
Steroid treatment	18 (22.2)	232 (38.0)	.005	991 (1.8)	<.001
Chorioamnionitis	5 (6.2)	25 (4.1)	.38	1 047 (1.9)	.02
Payment					
Medicaid	52 (64.2)	323 (53.0)	.14	30 969 (55.4)	.18
Private Insurance	17 (21.0)	170 (27.9)		15 157 (27.1)	
Self-Pay	8 (9.9)	51 (8.4)		4 233 (7.6)	
Other	4 (4.9)	66 (10.8)		5 535 (9.9)	
Other birth outcomes					
Five-minute APGAR score					
A score of 0-3	42 (51.9)	28 (4.6)	<.001	263 (0.5)	-
A score of 4-6	21 (25.9)	111 (18.2)		945 (1.7)	
A score of 7-8	12 (14.8)	299 (49.0)		8 138 (14.6)	
A score of 9-10	1 (1.2)	164 (26.9)		46 372 (83.0)	
Missing	5 (6.2)	8 (1.3)		176 (0.3)	
Final route and method of birth					
Spontaneous	43 (40.6)	227 (37.2)	.02	37 729 (67.5)	-
Forceps	1 (0.9)	3 (0.5)		410 (0.7)	
Vacuum	0 (0.0)	1 (0.2)		1 277 (2.3)	
Cesarean	37 (34.9)	379 (62.1)		16 473 (29.5)	
Missing	25 (23.6)	0 (0.0)		5 (0.0)	

NND, neonatal death. FAR, fetuses-at-risk. GA, gestational age. GWG, gestational age.

[a] Corresponding to censored cohorts which did not experience NND as described in the Methods.

[b] P-value was calculated compared to neonatal death group.

[c] GA 22-31 weeks indicates 22 0/7 weeks – 31 6/7 weeks.

[d] Missing data on labelled characteristics are not indicated but are below 0.5%.

© 2025 Wu B et al. *JAMA Network Open*.

[e] Since the data files does not have information for weight gain in each trimester, the rate of GWG was classified within pre-pregnancy BMI categories (described in the METHODS).

© 2025 Wu B et al. *JAMA Network Open*.

eTable 3. Unadjusted Hazard Ratios for Risk of Neonatal Death Following Preterm Birth (22-27 Weeks, 22-31 Weeks, 22-36 Weeks) Using Birth-Based Approach

	Hazard Ratio (95% CI)					
**Variable**	**22-27 weeks**	**P-value**	**22-31 weeks**	**P-value**	**22-36 weeks**	**P-value**
Individual						
Sex						
Female	1 [Reference]		1 [Reference]		1 [Reference]	
Male	1.43 (0.89-2.29)	.14	1.25 (0.80-1.95)	.32	1.40 (0.94-2.06)	.09
Maternal						
Ethnicity						
Hawaiian	1 [Reference]		1 [Reference]		1 [Reference]	
Guamanian	0.45 (0.11-1.78)	.25	0.48 (0.14-1.62)	.23	0.63 (0.22-1.81)	.39
Samoan	0.82 (0.30-2.21)	.69	0.82 (0.33-2.02)	.66	1.24 (0.54-2.85)	.61
Other Pacific Islanders	1.49 (0.64-3.46)	.36	1.07 (0.49-2.34)	.87	1.43 (0.69-2.97)	.33
Age						
< 20 years	1.15 (0.55-2.43)	.71	1.50 (0.74-3.03)	.26	1.70 (0.93-3.13)	.09
20-34 years	1 [Reference]		1 [Reference]		1 [Reference]	
>= 35 years	0.78 (0.43-1.44)	.43	0.86 (0.49-1.51)	.59	0.93 (0.56-1.53)	.77
Nativity						
Born in the US	1 [Reference]		1 [Reference]		1 [Reference]	
Born outside the US	0.94 (0.57-1.42)	.79	1.16 (0.73-1.83)	.54	0.95 (0.63-1.42)	.79
Marital status						
Married	1 [Reference]		1 [Reference]		1 [Reference]	
Unmarried	1.19 (0.72-1.95)	.50	1.07 (0.68-1.69)	.78	0.92 (0.62-1.36)	.67
Education						
Less than high school	1 [Reference]		1 [Reference]		1 [Reference]	
High school graduate	1.02 (0.58-1.78)	.95	0.97 (0.57-1.66)	.92	1.16 (0.73-1.83)	.54
Some college credit (no degree)	0.60 (0.29-1.21)	.15	0.91 (0.48-1.69)	.76	1.04 (0.61-1.78)	.89
Associate degree or above	0.68 (0.30-1.52)	.34	0.78 (0.36-1.69)	.54	0.64 (0.31-1.35)	.24
Death of prior children	2.58 (1.12-5.97)	.03	3.35 (1.46-7.69)	.004	3.59 (1.74-7.37)	<.001
Subsequent live birth	1.23 (0.77-1.96)	.39	1.01 (0.65-1.58)	.95	0.81 (0.55-1.21)	.30
Summary of Adequacy of Prenatal Care Utilization Index						
Inadequate	1.26 (0.59-2.69)	.56	1.01 (0.49-2.09)	.98	0.92 (0.51-1.64)	.77
Intermediate	1.18 (0.32-4.37)	.80	0.98 (0.27-3.56)	.97	0.52 (0.15-1.77)	.30
Adequate	1 [Reference]		1 [Reference]		1 [Reference]	

© 2025 Wu B et al. *JAMA Network Open*.

Adequate Plus	0.89 (0.42-1.84)	.74	0.91 (0.45-1.81)	.78	1.22 (0.71-2.12)	.47
Enrolled in WIC program	0.95 (0.59-1.55)	.85	0.88 (0.56-1.37)	.57	0.83 (0.56-1.22)	.34
Smoked before pregnancy	0.99 (0.90-1.09)	.87	0.99 (0.90-1.08)	.77	0.99 (0.46-2.13)	.97
Smoked during 1st trimester	0.99 (0.90-1.09)	.86	0.99 (0.90-1.08)	.76	1.09 (0.48-2.48)	.84
Smoked during 2nd trimester	0.98 (0.88-1.08)	.66	0.97 (0.87-1.09)	.61	0.88 (0.32-2.40)	.80
Smoked during 3rd trimester	1.05 (0.95-1.15)	.34	1.02 (0.93-1.12)	.66	0.87 (0.28-2.76)	.81
Body mass index						
Underweight (<18.5)	2.13 (0.60-7.55)	.24	1.94 (0.65-5.77)	.23	1.87 (0.64-5.45)	.25
Healthy weight (18.5-24.9)	1 [Reference]		1 [Reference]		1 [Reference]	
Overweight (25.0-29.9)	1.09 (0.53-2.27)	.81	0.89 (0.47-1.70)	.73	1.10 (0.62-1.94)	.74
Obesity I (30.0-34.9)	1.27 (0.62-2.60)	.51	1.24 (0.65-2.35)	.51	1.48 (0.84-2.59)	.17
Obesity II (35.0-39.9)	1.28 (0.58-2.85)	.55	1.29 (0.63-2.65)	.49	1.47 (0.76-2.86)	.25
Extreme Obesity III (>=40.0)	0.94 (0.35-2.50)	.90	0.88 (0.45-1.57)	.78	1.18 (0.54-2.57)	.68
Rate of GWG (lb/week, by stratified BMI)[b]						
< 10th percentile	0.82 (0.39-1.87)	.52	0.84 (0.45-1.57)	.59	1.09 (0.63-1.86)	.76
10th-90th percentile	1 [Reference]		1 [Reference]		1 [Reference]	
> 90th percentile	0.85 (0.39-1.87)	.68	1.10 (0.55-2.21)	.79	1.13 (0.62-2.07)	.70
Pre-pregnancy diabetes[c]	-	-	-	-	0.20 (0.03-1.43)	.11
Gestational diabetes	0.61 (0.19-1.95)	.41	0.38 (0.12-1.20)	.10	0.22 (0.07-0.69)	.002
Pre-pregnancy hypertension	0.19 (0.03-1.37)	.10	0.17 (0.02-1.21)	.08	0.21 (0.03-1.52)	.12
Gestational hypertension	0.90 (0.40-2.07)	.80	0.81 (0.41-1.62)	.55	0.76 (0.41-1.42)	.39
Hypertension eclampsia[c]	-	-	-	-	-	-
Previous preterm birth	1.84 (1.01-3.35)	.05	1.56 (0.89-2.74)	.12	1.33 (0.78-2.26)	.30
Steroid treatment	0.52 (0.29-0.96)	.04	0.49 (0.29-0.82)	.007	1.24 (0.76-2.01)	.39
Chorioamnionitis	1.33 (0.53-3.29)	.54	1.48 (0.60-3.66)	.39	3.48 (1.42-8.55)	.007
Payment						
Medicaid	1 [Reference]		1 [Reference]		1 [Reference]	
Private Insurance	0.68 (0.38-1.22)	.19	0.65 (0.37-1.12)	.12	0.69 (0.42-1.12)	.14
Self Pay	1.12 (0.53-2.37)	.77	0.97 (0.46-2.03)	.93	1.45 (0.84-2.50)	.19
Other	0.64 (0.23-1.77)	.39	0.40 (0.15-1.12)	.08	0.44 (0.18-1.08)	.07

[a] GA 22-27 weeks indicates 22 0/7 weeks – 27 6/7 weeks; GA 22-31 weeks indicates 22 0/7 weeks – 31 6/7 weeks; GA 22-36 weeks indicates 22 0/7 weeks - 36 6/7 weeks.
[b] Since the data files does not have information for weight gain in each trimester, the rate of GWG was classified within pre-pregnancy BMI categories (described in the **Appendix 1**).
[c] Some modelling outcome on labelled characteristics were not available due to a small number of cases.

© 2025 Wu B et al. *JAMA Network Open*.

eTable 4. Unadjusted Hazard Ratios for Risk of Neonatal Death Following Preterm Birth (22-27 Weeks, 22-31 Weeks, 22-36 Weeks) Using Fetuses-at-Risk Approach

	Hazard Ratio (95% CI)					
**Variable**	**22-27 weeks**	**P-value**	**22-31 weeks**	**P-value**	**22-36 weeks**	**P-value**
Individual						
Sex						
Female	1 [Reference]		1 [Reference]		1 [Reference]	
Male	1.30 (0.81-2.09)	.27	1.39 (0.89-2.16)	.15	1.51 (1.02-2.24)	.04
Maternal						
Ethnicity						
Hawaiian	1 [Reference]		1 [Reference]		1 [Reference]	
Guamanian	0.37 (0.09-1.49)	.16	0.43 (0.13-1.46)	.17	0.56 (0.19-1.61)	.28
Samoan	0.86 (0.32-2.32)	.76	0.94 (0.38-2.32)	.88	1.05 (0.46-2.42)	.91
Other Pacific Islanders	1.33 (0.57-3.10)	.51	1.25 (0.57-2.75)	.58	1.45 (0.70-3.00)	.32
Age						
< 20 years	2.01 (0.95-4.25)	.07	1.99 (0.98-401)	.06	2.01 (1.10-3.70)	.02
20-34 years	1 [Reference]		1 [Reference]		1 [Reference]	
>= 35 years	1.44 (0.78-2.65)	.24	1.46 (0.83-2.58)	.19	1.41 (0.85-2.32)	.19
Nativity						
Born in the US	1 [Reference]		1 [Reference]		1 [Reference]	
Born outside the US	1.11 (0.68-1.80)	.68	1.13 (0.72-1.79)	.60	1.04 (0.69-1.56)	.86
Marital status						
Married	1 [Reference]		1 [Reference]		1 [Reference]	
Unmarried	1.38 (0.84-2.26)	.21	1.25 (0.79-1.98)	.34	1.05 (0.71-1.56)	.82
Education						
Less than high school	1 [Reference]		1 [Reference]		1 [Reference]	
High school graduate	1.02 (0.59-1.79)	.94	1.00 (0.58-1.70)	.99	0.99 (0.63-1.58)	.98
Some college credit (no degree)	0.64 (0.32-1.31)	.22	0.83 (0.44-1.55)	.56	0.83 (0.49-1.43)	.51
Associate degree or above	0.62 (0.28-1.40)	.25	0.64 (0.30-1.38)	.25	0.47 (0.23-0.99)	.05
Death of prior children	5.61 (2.43-12.95)	<.001	4.87 (2.12-11.18)	<.001	4.97 (2.42-10.22)	<.001
Subsequent live birth	0.55 (0.35-0.89)	.01	0.62 (0.40-0.97)	.03	0.74 (0.50-1.09)	.13
Adequacy of Prenatal Care Utilization Index						
Inadequate	2.62 (1.22-5.62)	.01	2.45 (1.18-5.09)	.02	1.94 (1.09-3.47)	.02
Intermediate	1.00 (0.27-3.69)	1.00	0.90 (0.25-3.26)	.87	0.53 (0.16-1.80)	.31
Adequate	1 [Reference]		1 [Reference]		1 [Reference]	

© 2025 Wu B et al. *JAMA Network Open*.

Adequate Plus	5.85 (2.80-12.19)	<.001	6.50 (3.26-12.96)	<.001	4.65 (2.68-8.04)	<.001
Enrolled in WIC program	0.57 (0.35-0.92)	.02	0.64 (0.41-1.00)	.05	0.70 (0.47-1.03)	.07
Smoked before pregnancy	0.99 (0.91-1.07)	.75	0.98 (0.90-1.06)	.62	0.98 (0.92-1.05)	.63
Smoked during 1st trimester	1.01 (0.95-1.08)	.70	1.01 (0.94-1.08)	.83	1.01 (0.94-1.07)	.84
Smoked during 2nd trimester	1.01 (0.93-1.10)	.79	1.01 (0.92-1.10)	.90	1.00 (0.91-1.10)	.98
Smoked during 3rd trimester	1.04 (0.98-1.09)	.22	1.03 (0.97-1.09)	.31	1.02 (0.96-1.09)	.44
Body mass index						
Underweight (<18.5)	3.59 (1.01-12.73)	.05	3.38 (1.14-10.05)	.03	2.74 (0.94-7.98)	.06
Healthy weight (18.5-24.9)	1 [Reference]		1 [Reference]		1 [Reference]	
Overweight (25.0-29.9)	1.35 (0.65-2.80)	.42	1.06 (0.55-2.02)	.87	1.20 (0.68-2.11)	.53
Obesity I (30.0-34.9)	1.97 (0.97-4.04)	.06	1.46 (0.77-2.77)	.24	1.64 (0.93-2.87)	.09
Obesity II (35.0-39.9)	2.24 (1.01-4.98)	.05	1.71 (0.83-3.53)	.14	1.60 (0.82-3.10)	.16
Extreme Obesity III (>=40.0)	1.61 (0.60-4.28)	.34	1.13 (0.45-2.88)	.79	1.38 (0.63-3.01)	.42
Rate of GWG (lb/week, stratified by BMI)[b]						
< 10th percentile	1.84 (0.98-3.45)	.06	1.60 (0.86-2.97)	.14	1.64 (0.96-2.81)	.07
10th-90th percentile	1 [Reference]		1 [Reference]		1 [Reference]	
> 90th percentile	1.06 (0.48-2.32)	.89	1.18 (0.58-2.37)	.65	1.21 (0.66-2.21)	.55
Pre-pregnancy diabetes[c]	-	-	-	-	0.59 (0.08-4.24)	.60
Gestational diabetes	0.50 (0.16-1.58)	.24	0.43 (0.14-1.38)	.16	0.33 (0.10-1.04)	.06
Pre-pregnancy hypertension	0.93 (0.13-6.70)	.95	0.82 (0.11-5.85)	.84	0.62 (0.09-4.45)	.63
Gestational hypertension	1.58 (0.69-3.66)	.28	2.15 (1.07-4.29)	.03	1.99 (1.07-3.71)	.03
Hypertension eclampsia[c]	-	-	-	-	-	-
Previous preterm birth	5.35 (2.93-9.77)	<.001	5.43 (3.10-9.51)	<.001	4.25 (2.50-7.23)	<.001
Steroid treatment	12.24 (6.71-22.34)	<.001	15.63 (9.26-26.39)	<.001	12.76 (7.84-20.76)	<.001
Chorioamnionitis	3.96 (1.60-9.83)	.003	3.44 (1.40-8.51)	.007	2.59 (1.06-6.36)	.04
Payment						
Medicaid	1 [Reference]		1 [Reference]		1 [Reference]	
Private Insurance	0.70 (0.39-1.25)	.23	0.67 (0.39-1.16)	.15	0.67 (0.41-1.10)	.11
Self-Pay	1.33 (0.63-2.83)	.46	1.13 (0.54-2.37)	.76	1.83 (1.06-3.16)	.03
Other	0.51 (0.18-1.42)	.20	0.43 (0.16-1.19)	.10	0.44 (0.18-1.09)	.07

[a] GA 22-27 weeks indicates 22 0/7 weeks – 27 6/7 weeks; GA 22-31 weeks indicates 22 0/7 weeks – 31 6/7 weeks; GA 22-36 weeks indicates 22 0/7 weeks - 36 6/7 weeks.
[b] Since the data files does not have information for weight gain in each trimester, the rate of GWG was classified within pre-pregnancy BMI categories (described in the **Appendix 1**).
[c] Some modelling outcome on labelled characteristics were not available due to a small number of cases.

© 2025 Wu B et al. *JAMA Network Open*.

eTable 5. Birth Rates, Birth-Based Effect Estimates, and Fetuses-at-Risk Effect Estimates in US Pacific Islander Individuals (GA 22-27 Weeks).

	Neonates born with GA 22-27 weeks				
**Characteristics**	**Birth rate[b]**	**Ratio of birth rate**	**Birth-based aHR**	**FAR aHR**	**(Ratio of birth rate) x (birth-based aHR)**
Pre-pregnancy BMI					
Underweight (<18.5)	6.7961	1.93	1.67	3.24	3.23
Healthy weight (18.5-24.9)	3.5166	1 [Reference]	1 [Reference]	1 [Reference]	1 [Reference]
Overweight (25.0-29.9)	4.3148	1.23	1.26	1.50	1.55
Obesity Class I (30.0-34.9)	5.5240	1.57	1.33	2.31	2.09
Obesity Class II (35.0-39.9)	6.2037	1.76	1.73	2.82	3.05
Obesity Class III (>=40.0)	5.8657	1.67	1.41	2.18	2.35

GA, gestational age. aHR, adjusted hazard rate ratio. FAR, fetuses-at-risk. BMI, body mass index. Ref., reference.
[a] GA 22-27 weeks indicates 22 0/7 weeks – 27 6/7 weeks.
[b] Birth rate: birth per 1,000 fetus-weeks.

© 2025 Wu B et al. *JAMA Network Open*.

eTable 6. Birth Rates, Birth-Based Effect Estimates, and Fetuses-at-Risk Effect Estimates in US Pacific Islander Individuals (GA 22-31 Weeks).

	Neonates born with GA 22-31 weeks				
**Characteristics**	**Birth rate[b]**	**Ratio of birth rate**	**Birth-based aHR**	**FAR aHR**	**(Ratio of birth rate) x (birth-based aHR)**
Pre-pregnancy BMI					
Underweight (<18.5)	19.4175	1.84	1.74	3.16	3.20
Healthy weight (18.5-24.9)	10.5498	1 [Reference]	1 [Reference]	1 [Reference]	1 [Reference]
Overweight (25.0-29.9)	12.3974	1.18	1.04	1.15	1.22
Obesity Class I (30.0-34.9)	12.4890	1.18	1.53	1.63	1.81
Obesity Class II (35.0-39.9)	14.0717	1.33	2.03	2.06	2.71
Obesity Class III (>=40.0)	13.4695	1.28	1.33	1.43	1.70

GA, gestational age. aHR, adjusted hazard rate ratio. FAR, fetuses-at-risk. BMI, body mass index. Ref., reference.
[a] GA 22-31 weeks indicates 22 0/7 weeks – 31 6/7 weeks.
[b] Birth rate: birth per 1,000 fetus-weeks.

© 2025 Wu B et al. *JAMA Network Open*.

eTable 7. Birth Rates, Birth-Based Effect Estimates, and Fetuses-at-Risk Effect Estimates in US Pacific Islander Individuals (GA 22-36 Weeks, all PTB).

	Neonates born with GA 22-36 weeks				
**Characteristics**	**Birth rate[b]**	**Ratio of birth rate**	**Birth-based aHR**	**FAR aHR**	**(Ratio of birth rate) x (birth-based aHR)**
Pre-pregnancy BMI					
Underweight (<18.5)	126.2136	1.47	1.68	2.57	2.48
Healthy weight (18.5-24.9)	85.6157	1 [Reference]	1 [Reference]	1 [Reference]	1 [Reference]
Overweight (25.0-29.9)	93.3455	1.09	1.22	1.30	1.33
Obesity Class I (30.0-34.9)	94.7882	1.11	1.85	1.86	2.05
Obesity Class II (35.0-39.9)	93.0549	1.09	1.97	1.97	2.14
Obesity Class III (>=40.0)	100.1521	1.17	1.73	1.80	2.02

PTB, preterm birth. GA, gestational age. aHR, adjusted hazard rate ratio. FAR, fetuses-at-risk. BMI, body mass index. Ref., reference.
[a] GA 22-36 weeks indicates 22 0/7 weeks - 36 6/7 weeks.
[b] Birth rate: birth per 1,000 fetus-weeks.

© 2025 Wu B et al. *JAMA Network Open*.

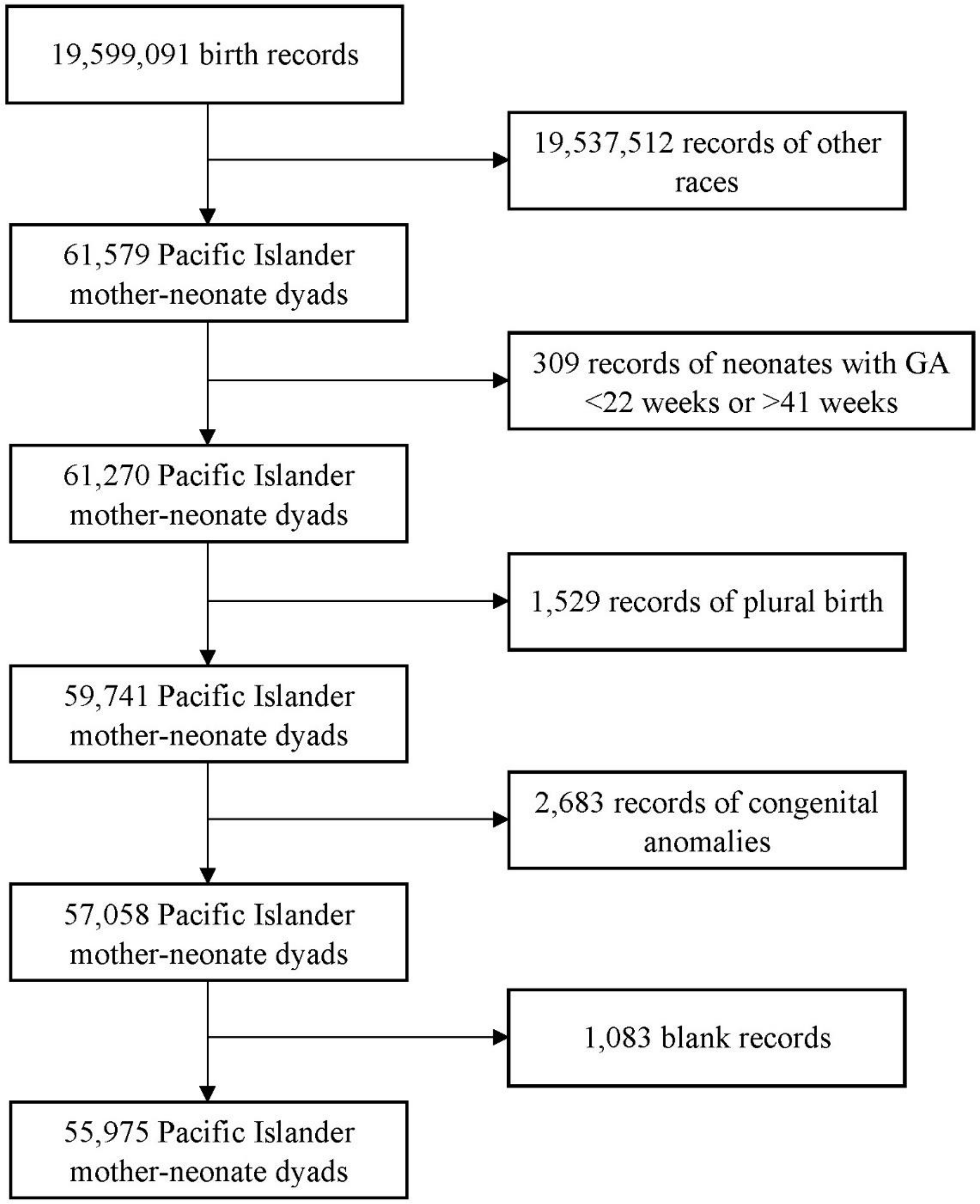

eFigure 1. Flowchart of the Sample Selection, 2014-2018 Birth Cohort Birth Linked Infant Death Data Files
GA, gestational age

© 2025 Wu B et al. *JAMA Network Open*.

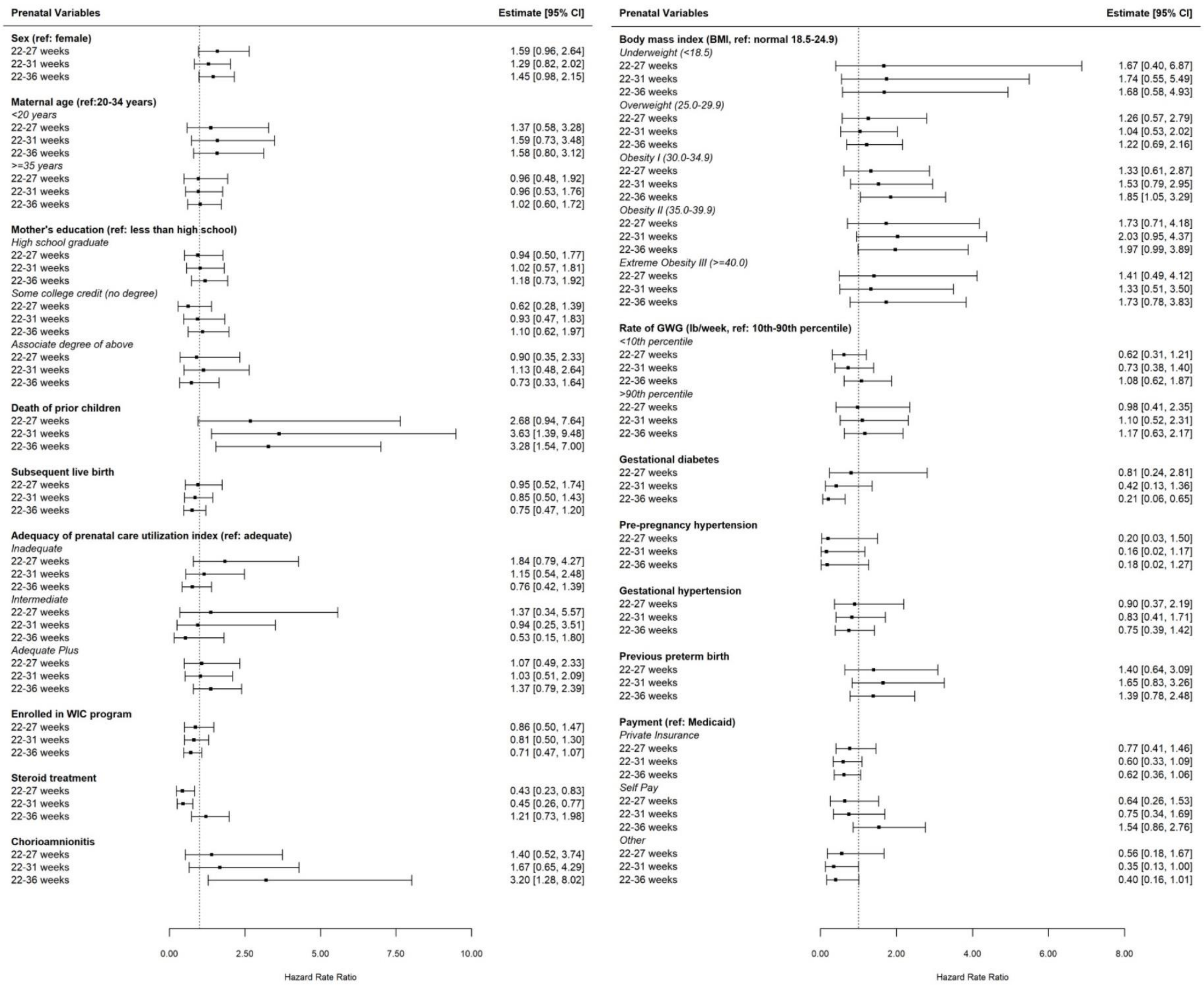

eFigure 2. Adjusted Hazard Ratios for Risk of Neonatal Death Following Preterm Birth Using the Birth-Based Approach

© 2025 Wu B et al. *JAMA Network Open*.

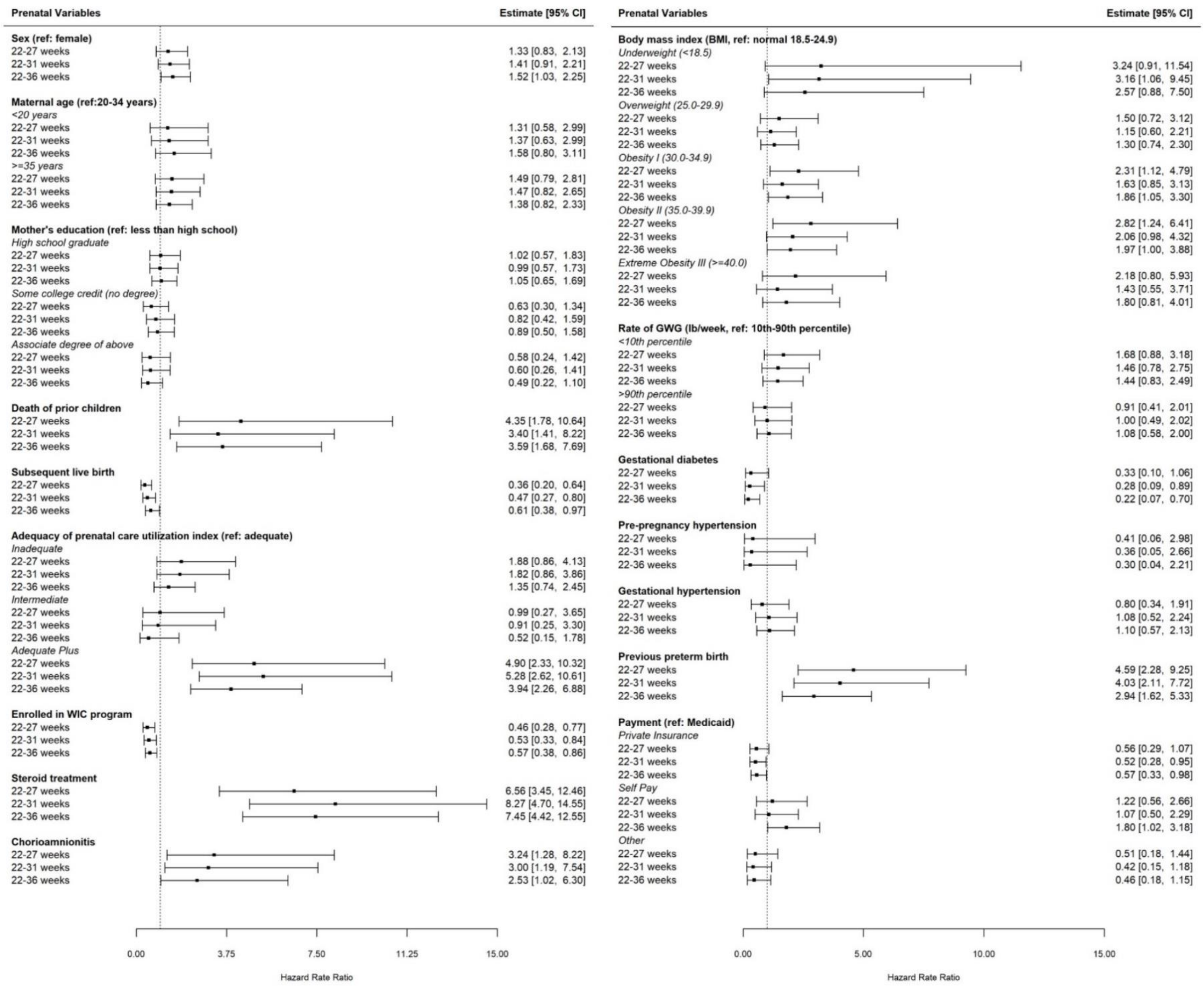

eFigure 3. Adjusted Hazard Ratios for Risk of Neonatal Death Following Preterm Birth Using the Fetuses-at-Risk Approach

© 2025 Wu B et al. *JAMA Network Open*.
