# Supplementary material for: Maternal Obesity and Neonatal Death in Preterm US Pacific Islander Neonates Using 2 Analytic Approaches
Source: JAMA Netw Open. 2025 Aug 26;8(8):e2528924. doi: 10.1001/jamanetworkopen.2025.28924 (PMC12381668; doi:10.1001/jamanetworkopen.2025.28924)
Supplement: Supplement 2. — Data Sharing Statement [file jamanetwopen-e2528924-s002.pdf]

Data Sharing Statement

Wu. Maternal Obesity and Neonatal Death in Preterm US Pacific Islander Neonates Using 2 Analytic Approaches. *JAMA Netw Open*. Published August 26, 2025.
doi:10.1001/jamanetworkopen.2025.28924

Data
Data available: Yes
Data types: Deidentified participant data
How to access data: This is a public dataset provided by the US National Center for Health Statistics. Data access: https://www.cdc.gov/nchs/data_access/vitalstatsonline.htm#Births.
When available: With publication

Supporting Documents
Document types: Other (please specify)
Additional Information: This is a public dataset provided by the US National Center for Health Statistics. Data access: https://www.cdc.gov/nchs/data_access/vitalstatsonline.htm#Births.
How to access documents: This is a public dataset provided by the US National Center for Health Statistics. Data access:
https://www.cdc.gov/nchs/data_access/vitalstatsonline.htm#Births.
When available: With publication

Additional Information
Who can access the data: This is a public dataset provided by the US National Center for Health Statistics. Data access:
https://www.cdc.gov/nchs/data_access/vitalstatsonline.htm#Births.
Types of analyses: This is a public dataset provided by the US National Center for Health Statistics. Data access: https://www.cdc.gov/nchs/data_access/vitalstatsonline.htm#Births.
Mechanisms of data availability: This is a public dataset provided by the US National Center for Health Statistics. Data access:
https://www.cdc.gov/nchs/data_access/vitalstatsonline.htm#Births.
